# Effects of extremely low-frequency electromagnetic field on expression levels of some antioxidant genes in human MCF-7 cells

**Published:** 2016-06

**Authors:** Hamideh Mahmoudinasab, Fatemeh Sanie-Jahromi, Mostafa Saadat

**Affiliations:** Department of Biology, College of Sciences, Shiraz University, Shiraz 71467-13565, Iran

**Keywords:** ELF-EMF, Antioxidant, Gene expression, MCF-7

## Abstract

In the past three decades, study on the biological effects of extremely low-frequency electromagnetic fields (ELF-EMFs) has been of interest to scientists. Although the exact mechanism of its effect is not fully understood, free radical processes has been proposed as a possible mechanism. This study was designed to evaluate the effect of 50-Hz EMFs on the mRNA levels of seven antioxidant genes (*CAT*, *SOD1*, *SOD2*, *GSTO1*, *GSTM3*, *MSGT1*, and *MSGT3*) in human MCF-7 cells. The EMF exposure patterns were: 1) 5 min field-on/5 min filed-off, 2) 15 min field-on/15 min field-off, 3) 30 min field-on continuously. In all three exposure conditions we tried to have total exposure time of 30 minutes. Control cultures were located in the exposure apparatus when the power was off. The experiments were done at two field intensities; 0.25 mT and 0.50 mT. The RNA extraction was done at two times; immediately post exposure and two hours post exposure. The mRNA levels were determined using quantitative real-time polymerase chain reaction. MTT assay for three exposure conditions in the two field intensities represented no cytotoxic effect on MCF-7 cells. Statistical comparison showed a significant difference between 0.25 mT and 0.50 mT intensities for "the 15 min field-on/15 min field-off condition" (Fisher's exact test, P=0.041), indicating that at 0.50 mT intensity field, the number of down-regulated and/or up-regulated genes increased compared with the other ones. However, there is no statistical significant difference between the field intensities for the two others EMF exposure conditions.

## INTRODUCTION

From the first report of relationship between cancer incidence and exposure to extremely low-frequency electromagnetic fields (ELF-EMFs) [1], the ELF-EMFs have been classified as a potential carcinogenic factor by International Agency for Research on Cancer (IARC) [2]. Therefore, many research groups have turned their attention to the different biological effects of the ELF-EMFs such as changes in development stages [3], genotoxic effects [4, 5], and alterations in gene expression [6-8]. However, there were some reports indicating that the EMFs have no biological effects [9, 10]. 

It is reported that elevation of reactive oxygen species (ROS) production and/or increasing the lifetime of ROS might be associated with mechanism(s) of the EMFs effects on biological systems [11-18]. It is well established that the elevation in concentration of ROS within cells causes oxidative stress. It is observed that oxidative stress involved in processes such as alteration in enzymes activity, gene expression, DNA damage, tumor initiation, tumor progression and neurodegenerative diseases [19-25]. Cells have especial enzymatic antioxidant defense systems against oxidative stress. These systems play a key role to protect cells from destructive free radicals activity. Catalase (CAT), superoxide dismutases (SODs) and glutathione S-transferases (GSTs) are enzymes of these systems [26, 27]. In the present study, we investigated whether ELF-EMFs could induce any changes in the mRNA level of seven antioxidant genes (*CAT*, *SOD1*, *SOD2*, *GSTO1*, *GSTM3*, *MSGT1*, and *MSGT3*). 

## MATERIALS AND METHODS

**Cell culture: **Human breast adenocarcinoma cell line MCF-7 was obtained from National Cell Bank of Iran (NCBI) (Pasteur Institute, Iran) and cultured in RPMI-1640 with L-glutamine, supplemented with 10% fetal bovine serum, 100 U/ml penicillin and 100 µg/ml streptomycin (all from Gibco). Cells were incubated in a tissue culture incubator at 37°C and 5% CO_2_. Approximately 24h before EMFs exposure, cells were seeded at the density of 3×10^5 ^cells/ml in 100 mm surface treated tissue culture Petri dishes.

**Electromagnetic field exposure system: **A solenoid with the 44 cm length and 14 cm diameter consisting of 200 turns of 10^-3^m diameter copper wire wounded in two layers, and working with 50-Hz sinusoidal alternating current was used as electromagnetic apparatus. The solenoid was placed in a box shielded with 2 layers of aluminum and 1 layer of copper. Current intensity was set to the desired level by means of an electric rotary converter (Emersun). Field density is calculated by B = µ_0_NI/L formula, where B is field density (T), µ_0 _is the vacuum permeability and equals to 4π × 10^-7^ (N/A^2^), N is the number of turns, I is the current in the wire (A) and L is the solenoid length (m). Field work accuracy was calibrated by means of an EMF tester (Lutron electronic enterprise Co.) at the beginning of each test. Cell culture dishes were located on the axis of the solenoid to be exposed almost uniformly. 

**Exposure conditions: **Three conditions of exposure were designed; two intermittent and one continuous. In all three exposure conditions we tried to have total exposure time of 30 minutes. The EMF exposure conditions were: 1) 5 min field-on/5 min filed-off, 2) 15 min field-on/15 min field-off, 3) 30 min field-on continuously. Control cultures were located in the exposure apparatus when the power was off. Temperature changes were monitored during exposure time and no change was observed. The experiments were done at two field intensities; 0.25 mT and 0.50 mT. Control Petri dishes for each of three conditions were kept in disconnected solenoid for an equal time to EMF exposure. The RNA extractions were done at two times; immediately post exposure (0h) and two hours post exposure (2h). 

**MTT assay: **Cell viability was measured by MTT (3-[4,5-dimethylthiazol-2yl] diphenyltetra-zolium bromide)) assay (Roche). The MCF-7 cells were seeded at a density of 1.5×10^4 ^cells/well in 96-well microplates (Jetbiofil). After cells exposed to ELF-EMF, plates returned to incubator and after 24h, 10 µl of MTT (Roche, 5 mg/ml in phosphate buffered saline) was added to each well. Microplates were incubated at 37°C for approximately 3h and then 100 µl of solubilization solution (10% SDS in 0.01 M HCl) were added to dissolve the formazan crystals and then incubated at 37°C overnight. After obtaining the absorption (at 545 nm), inhibition percentage of cell growth was calculated. MTT assay for each exposure condition was done in triplicate.

**RNA extraction, cDNA synthesis and Real-time RT-PCR: **Total RNA extractions were performed at the times of 0 and 2hr post exposure, using RNX-plus kit (Cinnagen Co., Iran) according to the manufacturer's protocol and then reverse transcribed to cDNA pool using primerscript^TM^ RT reagent kit (Takara Bio Inc., Japan) in a mixture of oligo-dT and random hexamer primers in accordance with the provider's instructions. The quality of extracted RNA was assessed by optical density (260/280nm ratios) and the concentration of the RNAs was measured by optical density at 260 nm. All samples had high quality of RNA (OD_260/280_=1.8-2.1). Primers specific for the studied genes and TATA box-binding protein (*TBP*, OMIM: 600075; used as a reference gene) were designed using Allele ID software (v.7.5, Premier Biosoft International, Palo Alto, CA, USA). The primer sequences are shown in Table 1. 

**Table1:** Sequences of applied primers and product size in real time PCR analysis

**Genes**	**OMIM**	**Forward 5’→3’**	**Reverse 5’→3’**	**Produ ** **ct size (bp)**
***TBP***	600075	CCCGAAACGCCGAATATAATC	T CT GGACT GTT CTTCACTCTT G	134
***CAT***	115500	CGGAGATTCAACACT GCCAAT G	TTCTT GACCGCTTTCTTCT GGA	155
***SOD1***	147450	GAAGGT GT GGGGAAGCATTAAAG	CAAGT CT CCAACAT GCCT CT CT	166
***SOD2***	147460	T GGGGTT GGCTT GGTTTCAA	GGAAT AAGGCCT GTT GTT CCTTG	95
***GSTO1***	605482	TGAAGTTAAAT GAGT GT GT AGACCA	CCTCAGGGCT GTTCT GT AAGT	147
***GSTM3***	138390	GACTTTCCT AAT CTGCCCT ACCT C	TTCTTCTTCAGT CT CACCACACAT	114
***MGST1***	138330	T GTACGCAGAGCCCACCT	GT AGAT CCGT GCT CCGACAAATAG	136
***MGST3***	604564	CCACCAGAACACGTTGGAAGT	GCT CCT CGACT ACGCTT GC	168

The primers were specific for mRNAs and could not amplify genomic DNA. Quantitative real time PCR analysis was carried out using SYBR^®^ premix Ex Taq^TM^ II (Takara Bio Inc., Japan) in Rotor-Gene 6000 instrument (Corbett research). A pre-amplification denaturation was performed at 95°C for 30 sec, followed by a two-step 

real-time PCR with a thermal profile that included 40 cycles of denaturation at 95°C for 5 sec, annealing and extension at 60°C for 45 sec, melting curve analysis was performed ramping from 75 to 95°C and rising 1 degree each step to confirm the precision of PCR reaction. Relative gene expression was calculated according to the 2^-ΔΔCt^ method based on the threshold cycle (Ct) values. We failed to investigate the alterations of *GSTT1*, *GSTM1*, and *GSTP1* because the MCF-7 cells have null genotypes and/or DNA methylation [28-30].

**Statistical analysis: **Data are shown as the mean ± SD of three independent experiments. Effects of exposure conditions on the mRNA levels of the examined genes were investigated using Analysis of Variance (ANOVA) followed by Bonferroni post hoc test. Fisher's exact test was used for the comparison between EMF intensities and the number of down-regulated or up-regulated genes. Statistically significant differences were assessed using student *t*-test by SPSS Statistical Package (SPSS Inc., Chicago, IL, USA) (version 11.5). A probability of P<0.05 was considered statistically significant.

## RESULTS

MTT assay for three exposure conditions in two field intensities (0.25 mT and 0.50 mT) represented no cytotoxic effect on MCF-7 cells (data not shown). Also, no changes in the morphology of cells were detected under microscope after each exposure condition. 

Table 2 showed the mRNA alterations of the examined genes after MCF-7 cells were exposed to 0.25 mT electromagnetic field. Based on statistical analysis (ANOVA), exposure conditions had significant effects on the mRNA levels of *SOD2* and *MGST3*. Post hoc test revealed that in the cells exposed to 30 min field-on continuously EMF the mRNA levels of *SOD2* (at 0h) and *MGST3* (at 2h) significantly decreased compared with unexposed cells. However, the expression of *MGST3* significantly increased (at 2h) when cells exposed with 5 min field-on/5 min filed-off of EMF.

Table 3 showed the mRNA alterations of the examined genes after MCF-7 cells were exposed to 0.50 mT intensity electromagnetic field. The analysis of variance indicating that exposure conditions had significant effects on the mRNA levels of *SOD2*, *GSTO1*, *GSTM3*, *MGST1* and *MGST3*. Post hoc test revealed that in the cells exposed to 30 min field-on continuously EMF the mRNA levels of *GSTM3* (at 2h) and *MGST3* (at 0h) significantly increased and decreased compared with unexposed cells, respectively. At the 15 min field-on/15 min field-off condition, *GSTO1* (at 2h) and *GSTM3* (at both 0h and 2h) decreased; whereas, *SOD2* (at 0h) and *MGST1* (at 2h) increased compared with the control levels. The 5 min field-on / 5 min filed-off condition, results in decreasing the mRNA levels of *GSTO1* (at 2h) and *GSTM3* (at 0h) compared with the unexposed cells.

Statistical comparison showed a significant difference between 0.5 mT and 0.25 mT intensities for "the 15 min field-on/15 min field-off condition" (Fisher's exact test, P=0.041), indicating that at 0.5 mT intensity field, the number of down-regulated and/or up-regulated genes increased compared with 

the other ones. However, there is no statistical significant difference between the field intensities for "the 30 min field-on continuously condition" and "the 5 min field-on/5 min field-off condition" (Fisher's Exact Test, P=1.0).

**Table 2:** mRNA levels (mean±SD) of some antioxidant genes in MCF-7 ce lls after e xposure to 50-Hz 0.25 mT ELF-EMFs

**Genes**	**Times afte r ** **exposure**	**Exposure conditions**			**Results of** **ANOVA**	
		**30 min cont.**	**15 On/15 Off**	**5 On/5 Off**	**F** **	**P**
***CAT***	0h	0.96± 0.11	1.03± 0.11	0.88± 0.15	1.11	0.399
	2h	1.14± 0.07	1.21± 0.19	1.12± 0.19	1.19	0.372
***SOD1***	0h	0.77± 0.15	0.80± 0.30	1.33± 0.19	5.29	0.027
	2h	1.05± 0.16	1.55± 0.27	1.04± 0.26	4.95	0.031
***SOD2***	0h	**0.59 ± 0.02** *	0.75 ± 0.17	1.06 ± 0.17	9.76	0.005
	2h	0.70 ± 0.14	0.99 ± 0.24	1.14 ± 0.25	2.96	0.067
***GSTO1***	0h	0.92± 0.15	0.70± 0.21	1.08± 0.15	3.64	0.064
	2h	1.18± 0.12	0.92± 0.19	0.90± 0.23	1.91	0.207
***GSTM3***	0h	1.00± 0.21	0.72± 0.14	0.83± 0.20	2.19	0.166
	2h	1.10± 0.08	0.86± 0.14	0.97± 0.26	1.34	0.328
***MGST1***	0h	0.92 ± 0.18	0.89 ± 0.22	0.74 ± 0.13	1.49	0.288
	2h	0.97 ± 0.05	0.93 ± 0.29	0.89 ± 0.17	0.22	0.875
***MGST3***	0h	0.83 ± 0.16	1.22 ± 0.11	1.28 ± 0.13	9.65	0.005
	2h	**0.60 ± 0.05** *	0.83 ± 0.10	**1.20 ± 0.06** *	48.1	<0.001

* P<0.05 all values compared with untreated controls (=1) using Bonferroni post hoc test.

** df=2, 8

**Table 3:** mRNA levels (mean + SD) of some antioxidant genes in MCF-7 ce lls after e xposure to 50-Hz 0.50 mT ELF-EMFs

**Genes**	**Times afte r ** **exposure**	**Exposure conditions**			**Results of ANOVA**	
		**30 min cont.**	**15 On/15 Off**	**5 On/5 Off**	**F** **	**P**
***CAT***	0h	0.92 ± 0.15	0.77 ± 0.13	0.87 ± 0.14	1.87	0.212
	2h	0.96 ± 0.14	0.95 ± 0.22	1.05 ± 0.21	0.23	0.870
***SOD1***	0h	0.73 ± 0.07	0.92 ± 0.01	1.18 ± 0.22	7.50	0.010
	2h	0.91 ± 0.06	1.02± 0.02	1.01 ± 0.09	2.48	0.135
***SOD2***	0h	1.17 ± 0.11	**1.36 ± 0.12** *	0.86 ± 0.18	9.61	0.005
	2h	1.20 ± 0.14	1.13 ± 0.11	0.85 ± 0.02	8.66	0.007
***GSTO1***	0h	0.81 ± 0.17	0.83 ± 0.22	0.67 ± 0.06	2.73	0.114
	2h	1.12 ± 0.08	**0.86 ± 0.04** *	**0.77 ± 0.06** *	21.9	<0.00 1
***GSTM3***	0h	0.79 ± 0.12	**0.65 ± 0.05** *	**0.69 ± 0.13** *	8.83	0.006
	2h	1.38 ± 0.10*	**0.72 ± 0.09** *	0.82 ± 0.10	33.2	<0.00 1
***MGST1***	0h	1.08 ± 0.15	1.33 ± 0.13	0.91 ± 0.19	5.31	0.026
	2h	1.03 ± 0.06	**1.36 ± 0.12** *	1.09 ± 0.06	16.1	0.001
***MGST3***	0h	**0.64 ± 0.06** *	1.00 ± 0.15	1.08 ± 0.11	12.7	0.002
	2h	1.14 ± 0.12	0.92 ± 0.16	0.82 ± 0.09	4.42	0.041

* P<0.05 all values compared with untreated controls (=1) using Bonferroni post hoc test.

** df=2, 8

## DISCUSSION

The exact mechanism of EMF within cells is not known. But more ROS has been observed after exposure to ELF-EMFs by several research groups [11-15]. To our knowledge, the present study is the first to evaluate the mRNA levels of some antioxidant genes following the MCF-7 cells exposed to the ELF-EMFs. During the day people can be exposed to intermittent EMFs, so we designed two modes of intermittent and one continuous exposure in our study. In two intermittent conditions the total time of exposure to ELF-EMFs is equal to continuous condition (30 minutes). 

There are some reports described the alterations in enzyme activities of SOD and CAT after cells or humans exposed to ELF-EMFs. The results were not consistent [8, 20, 31-36]. It may conclude that at least in part, the frequency of EMFs, intensity of the field, exposure times, and cell types, account for these differences. 

The current experiment was designed to establish whether ELF-EMFs might affect on mRNA levels of several genes involved in antioxidant pathways. Our present data indicated that the EMFs had some effects on the alterations of antioxidant genes. These alterations maximally seem at "the 15 min field-on/15 min field-off condition". Further studies on the enzyme activities of the examined genes and even the amount of their protein after exposure might reveal the effects of ELF-EMFs on oxidative stress to define precautions against oxidative damages on cells.
